# Well-being and mental stress in the population study of women in Gothenburg, Sweden: cohort comparisons from 1980 to 2016 of 36-year trends and socioeconomic disparities in 38-and 50-year old women

**DOI:** 10.1186/s12889-021-10937-z

**Published:** 2021-05-17

**Authors:** M. Waller, L. Lissner, D. Hange, V. Sundh, A. Blomstrand, C. Björkelund

**Affiliations:** 1 Primary Health Care, School of Public Health and Community Medicine, Institute of Medicine, Sahlgrenska Academy, University of Gothenburg, Box 453, 405 30 Gothenburg, Sweden; 2 Nutritional Epidemiology, School of Public Health and Community Medicine, Institute of Medicine, Sahlgrenska Academy, University of Gothenburg, Gothenburg, Sweden

**Keywords:** Well-being, Mental stress, Socioeconomic position, Women, Trends over time, Birth cohort comparisons

## Abstract

**Background:**

Women’s lives have dramatically changed in recent decades as evidenced by trends in educational attainment, employment outside the home, income, and other socioeconomic factors. Self-reported health in 18–70 year old women has been reported to be significantly lower than in men. In Sweden, the 2005 National Public Health Report showed that stressful work environments have become more common, especially for women. The purpose of the study was to monitor trends in well-being and perceived mental stress in the populations of 38- and 50-year-old women and to examine associations with socioeconomic position (SEP).

**Subjects:**

In 1980, 2004, and 2017, population-based samples of 38- and 50-year old women were recruited into the Prospective Population Study of Women in Gothenburg (PPSWG), Sweden. This population-based study included participants from selected birth cohorts to participate in health examinations, at similar ages and with similar protocols on each occasion.

**Methods:**

Birth cohort comparisons between three representative samples of 38- and 50-year-old women. Well-being (scale 1–7) and perceived mental stress (scale 1–6) based on questionnaires were the main outcomes studied in relation to time. Socioeconomic position (SEP) based on socio-occupational group, i.e. occupational and educational level combined, were examined as correlates of well-being and mental stress at different points in time.

**Results:**

Perception of good well-being increased in generations of 50-year-old women between 1980 to 2016, but no significant time trends were seen in 38-year-old women. Perception of high mental stress increased between 1980 and 2016, for both 38-and 50-year-old women. Belonging to a low socio-occupational group was associated with lower perceived well-being in 1980 but not in 2016. Belonging to a low socio-occupational group was not associated with perceived mental stress at any examination.

**Conclusions:**

Contemporary women of today have generally higher perceptions of well-being but also higher mental stress regardless of belonging to low or high socio-occupational group. Associations between poor well-being and belonging to a low socio-occupational group that were observed in 1980 and 2004 were not observed in 2016.

The Prospective Population Study of Women in Gothenburg, Sweden was approved by the ethics committee of University of Gothenburg (Dnr 65–80; Ö564–03; 258–16). The studies comply with the Declaration of Helsinki and informed consent has been obtained from the subjects.

## Background

Women’s life situations have dramatically changed in recent decades, in terms of education, employment, and own income. In a large WHO survey in 2004, data from 57 countries showed that self-reported health in 18–70 year old women was significantly lower than in men at all ages [1]. In Sweden, improvements in women’s health have been documented in the National Public Health Report from 2005 [2], which showed that female life expectancy has increased by 2 years since 1990. However, social disparities in life expectancy increased in 1986 to 2007 in Sweden, especially among women [3]. The same report also showed that stressful work environments have become more common especially for women. This is most prevalent in public sector workplaces such as healthcare, nursing and teaching, all of which predominantly employ women. In Gothenburg, Sweden, an increasing proportion of the female population state that they experience anxiety and mental stress, around 75% in 2004, compared to 25% in the 1960’s [4]. During a similar time period it was reported that the stress-levels in 50-year-old men were unchanged and continuously low, 17% [5].

In 1974, Sweden was the first Nordic country to introduce the law on parental insurance for both women and men [6], which greatly facilitated return to work outside the home after the mother’s parental leave. Another major change in women’s life situation in Sweden is the increasing mean age at first birth, which has risen from 24 years in 1980 to 28.5 years in 2006 and 28.6 in 2016 [7]. These changes have resulted in a transformation of the Swedish family in modern society [6].

In recent decades, a shift has also been observed in women’s perceived moods. In earlier generations of middle-aged women, family conditions were associated with women’s perceived mood, whereas for later generations, her situation at work was most significantly associated with their perceptions of mood [8]. Women’s work situation itself is greatly changed [9]. Increased educational opportunities and changing economic resources have affected employment possibilities for middle-aged women, resulting in changes in their employment rates. In the 1970’s less than 50% of women had full-time employment, compared to 75% in 2016. Corresponding rates of full time employment among men was 92% [10].

Perceived health, well-being and mental stress are closely related factors but the associations are complex and may have bi-directional components [11]. Moreover, reported associations between perceived stress on health and mortality [12] may be dependent on the type of stress and individual responses to stress. Given the changing lives and lifestyles of contemporary women, it is of particular interest to distinguish trends in well-being from trends in perceived stress and to view both factors in the context of socioeconomic conditions.

In the Prospective Population Study of Women in Gothenburg, three population-based samples of middle-aged women (38- and 50-year-olds) have participated in physical examinations with questionnaires on lifestyle in order to document secular trends in cardiovascular health indicators [13, 14]. These samples were recruited in 1980, 2004 and 2016, based on specific dates and years of birth, and answered identical questions on their well-being and perceived stress. Several cardiovascular risk factors related to lifestyle, i.e. smoking, blood lipid levels, and blood pressure levels, were improved in the middle-aged women in the latest decades [14]. A study concerning association between physical activity and well-being showed associations between high physical activity levels and increased well-being in women [15]. Trends concerning well-being in middle-aged women have also been studied in other populations [16]. However, relationships between socioeconomic position, well-being and perceived mental stress have been studied less frequently, and it is not known whether the trends are uniform in different socioeconomic groups of women over time.

### Aim

The aim of this study was to examine trends in well-being and perceived mental stress and the importance of socioeconomic position (SEP) in the populations of 38- and 50-year-old women in 1980, 2004 and 2016, respectively.

## Methods

### Design and setting

**Fig. 1:**
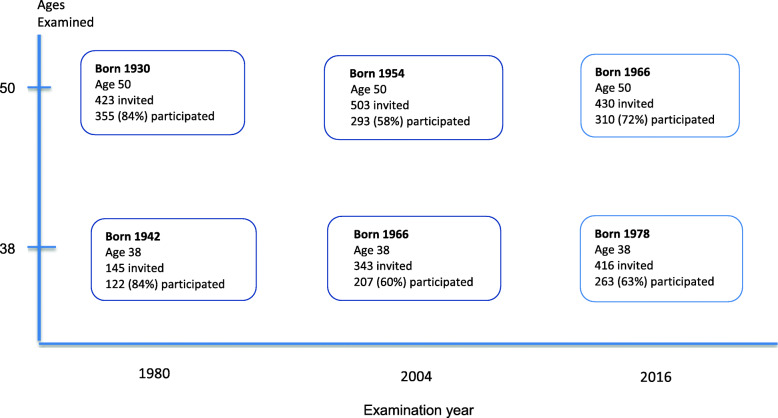
Description of the six 38- and 50-year-old birth cohorts examined in the Population Study of Women examinations in 1980, 2004 and 2016, respectively. Year of birth, number of invited women and number of women who participated in the respective examinations. Note that time axis reflects 24 and 12- year time intervals respectively between surveys

From the Population Study of Women in Gothenburg, Sweden [13, 14], we retrieved data on population-based representative samples of 38- and 50-year-old women who were examined in 1980, 2004 and in 2016, respectively (Fig. 1). The study population in 1980 (*n* = 477) comprised 38-year-old women born in 1942 (*n* = 122) and 50-year-old women born in 1930 (*n* = 355). The study population in 2004 (*n* = 500) consisted of 38-year-old women who were born in 1966 (*n* = 207) and the 50-year-old women were born in 1954 (*n* = 293) [14]. The study population in 2016 (*n* = 573) included 38-year-old women born in 1978 (*n* = 263) and 50-year-old women born in 1966 (*n* = 310). Of the 50-year-old women in 2016, 50% (155 women) had previously participated in the 2004 examination as 38-year-olds [14].

#### Well-being

Before taking part in the examination at the study premises, the participants were asked to answer a questionnaire on current well-being, family situation, social and educational status.

**Fig. 2:**
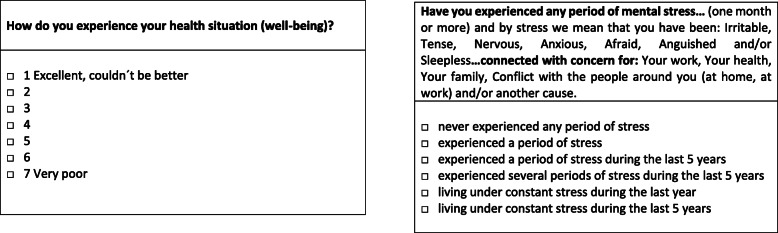
Questionnaire items concerning well-being and mental stress and their response categorisation in the Population Study of Women in Gothenburg

The question describing general well-being (Fig. 2) was: “How do you experience your health situation (well-being)?”. The answers were stated on a Likert-type scale from 1 to 7, where 1 was “excellent, couldn’t be better” and 7 was “very poor”. Good well-being was defined as categories 1 to 3. Poor well-being was defined as categories 4 to 7. When they answered this question, subjects were instructed that health situation/well-being reflects mental and physical health but no specific examples were given.

The question originates from the Gothenburg Quality of Life Instrument (GQL instrument) [17] developed in the 1960’s, based on World Health Organization, WHO’s definition of health as” a state of complete physical, mental and social well-being and not merely the absence of disease or infirmity” [18]. This question was evaluated in a study [19] and shown to have high reliability. Self-estimated well-being reflected both physical and psychological well-being [19]. A further evaluation of this well-being indicator was done for the present study. 1992 was the first time in the Population Study of Women examinations that the SF-36 and the original well-being indicator were used simultaneously. Coefficient of 0.69 between these two general well-being questions indicated high relative validity.

#### Mental stress

The women were asked to identify level of perceived mental stress based on a questionnaire item with the same wording throughout all examinations, which were given by the examining study physician. In the definition of mental stress, sleep problems were included as one of the symptoms of stress. The different categories are shown in Fig. 2. This question has previously been evaluated in another study and reliability was confirmed [20]. It has also been used in other prospective studies and the perceived highest levels of stress have been shown to be associated with risk of stroke and myocardial infarction [21].

Low mental stress was defined as levels “never experienced any period of stress”, “experienced a period of stress” and “experienced a period of stress during the last 5 years”, and all higher levels were defined as moderate and high mental stress in accordance to Fig. 2.

#### Time

The main exposure variable in the present study is time. The different birth cohorts of women were examined in 1980, 2004 or 2016.

#### Socioeconomic position

Socioeconomic position (SEP) was defined in terms of the social and economic factors that influence the positions that participants hold within the structure of society [22]. For the present study, socioeconomic position (SEP) was based on socio-occupational group, i.e. occupational and educational level combined, as described below.

The participants’ occupations were categorised into low, medium and high occupation, according to Carlsson’s standard occupations grouping system [23]. The variable was then dichotomised as low and medium-high. This categorisation was accomplished in accordance with the Swedish socioeconomic index [24], a broadly established socio-occupational classification method that includes the individual’s educational level [24]. SEP is not a biological “risk factor” but can be considered more of an environmental risk factor.

#### Other characteristics

Women were grouped, according to their smoking habits, as (yes) current smokers and (no) non-smokers and ex-smokers. BMI, body mass index (kg/m^2^) was based on measured weights and heights. The participants were then dichotomised in those having BMI < 25 and those ≥25. The question on leisure activity was based on the method described by Saltin and Grimby [25]. Women were interviewed by a physician and divided into physical activity groups according to the extent of their activity. Four groups: (1) low physical activity, being almost totally inactive; (2) intermediate, indicating some physical activity for at least 4 h per week; (3) high, meaning regular physical activity and (4) very high, hard physical activity and competition, were then dichotomised into low (1) and high (2-4).

### Statistics

Binary logistic models with outcome variables poor well-being and high stress were used to test for association with exposure factors time, age and SEP. Results are presented as odds ratios (OR) with 95% confidence intervals (CI). Associations are considered statistically significant at *p* < 0.05. Results were repeated in the full sample after stratification by SEP, with adjustment for age. Formal test of interaction was also conducted with regard to SEP on time trend (linear) in prevalence of poor well-being and high mental stress and presented as ORs. Software used in the analyses was SAS (Statistical Analysis System) version 9.4, Stata version 13 and 14.

## Results

### Descriptive data

The three different samples of 38- and 50-year-old women examined in 1980, 2004 and 2016 are presented in Table 1 regarding number of participants, well-being (poor/good), perceived mental stress level (low/medium-high), socio-occupational level (low/medium-high), smoking (yes/no), BMI (< 25/ ≥ 25) and leisure time physical activity (low/high). *P* values refer to changes over time.

**Table 1:** Characteristics of 38- and 50-year-old women in the Population Study of Women in Gothenburg regarding the assessments performed in 1980, 2004 and 2016, respectively (*n* = 1550). *P* values for linear trend over time

	1980		2004		2016			
	38 years 122 n (%)	50 years 355 n (%)	38 years 207 n (%)	50 years 293 n (%)	38 years 263 n (%)	50 years 310 n (%)	*P* 38 years	*P* 50 years
Well-being poor (4-7)	46 (38)	161 (46)	48 (24)	101 (35)	92 (35)	109 (36)		
Well-being good (1-3)	76 (62)	193 (54)	152 (76)	188 (65)	168 (65)	197 (64)	0.69	<0.01
Mental stress								
Low	95 (78)	293 (83)	109 (55)	134 (47)	94 (36)	157 (51)		
Medium-high	27 (22)	62 (17)	90 (45)	154 (53)	167 (64)	152 (49)	<0.01	<0.01
Socio-occupation								
Low	51 (43)	143 (47)	52 (26)	84 (29)	41 (17)	73 (24)		
Medium	57 (48)	129 (43)	100 (49)	145 (51)	118 (48)	145 (48)		
High	11 (9)	30 (10)	52 (25)	57 (20)	85 (35)	82 (27)	<0.01	<0.01
Smoking								
Yes	46 (38)	139 (39)	23 (11)	67 (23)	23 (9)	37 (12)		
No	76 (62)	216 (61)	181 (89)	224 (77)	240 (91)	273 (88)	<0.01	<0.01
BMI								
< 25	100 (83)	209 (59)	136 (69)	173 (62)	179 (68)	177 (58)		
≥ 25	21 (17)	146 (41)	60 (31)	108 (38)	83 (32)	131 (42)	0.01	0.77
Leisure time physical activity								
Low (1)	42 (34)	114 (32)	46 (22)	48 (17)	18 (7)	31 (10)		
High (2-4)	80 (66)	241 (68)	158 (78)	241 (83)	244 (93)	278 (90)	<0.01	<0.01

### Trends in well-being

In 38-year-old women, no significant differences were seen between those born in 1942, 1966 or 1978 regarding well-being (*p* = 0.69) (Table 1). In 50-year-old women, significant differences were seen between the three birth cohorts born in 1930, 1954 and 1966, where the trend showed an increased proportion of women who perceived their well-being as good (*p* < 0.01) (Table 1). Table 2 shows results from a fully adjusted model (all women) with time, age and SEP as separate predictors. Significant differences were found (*p* < 0.01), where the lower socio-occupational group were at excess risk of reporting poor well-being.

**Table 2:** Time, age and socio-economic position as separate predictors for poor well-being and high mental stress in all 38- and 50-year-old women (dependent variable). Logistic regression. Odds ratios (OR) with 95% confidence intervals (CI)

	Poor well-being OR (CI)	p	High mental stress OR (CI)	p
Time	0.91 (0.73–1.13)	0.41	2.38 (1.89–3.00)	**< 0.01**
Age	1.45 (0.90–1.49)	**0.05**	0.80 (0.61–1.05)	0.11
SEP	2.00 (1.31–3.04)	**< 0.01**	1.02 (0.76–1.37)	0.89

### Trends in mental stress

Concerning perceived mental stress, a significant trend of increased high mental stress (*p* < 0.01) was seen for both 38- and 50-year-old groups of women (Table 1). Table 2 shows that this was the trend for all women irrespective of age and SEP.

### Importance of SEP

Age-stratified associations between well-being/mental stress and socioeconomic position.

Table 3 shows associations between poor well-being (outcome variable) and low SEP in 1980, 2004 and 2016. In 1980 and 2004 there were significant associations (*p* = 0.05 and *p* < 0.01) between poor well-being and low SEP but not in 2016. Low socio-occupation as a risk factor for poor well-being was attenuated to non-significance in 2016. Table 3 also shows associations between high mental stress (outcome variable) and low SEP, in 1980, 2004 and 2016. No significant associations were found. Formal tests of interactions in all women, examining whether the risk factor low socio-occupational group affected the development of well-being over time and of mental stress over time. No significant interactions were found (*p* = 0.30 and 0.32). Thus, despite weak evidence that the relation between SEP and well-being was attenuated over time, no statistically significant modification by SEP of trends in well-being and mental stress could be observed.

**Table 3:** Test of the association between poor well-being (scale 4–7) (outcome)/ high mental stress (outcome), and low socio-occupational group, defined as the exposed group, 1980, 2004 and 2016. Logistic regression. Odds ratios (OR) with 95% confidence intervals (CI)

	Low socio-occupational group					
	1980		2004		2016	
	OR (CI)	*p*	OR (CI)	*p*	OR (CI)	*p*
Poor well-being scale 4-7						
38- and 50-year-olds	1.48 (1.00-2.19)	**0.05**	2.14 (1.41-3.24)	**<0.01**	1.19 (0.76-1.86)	0.46
High mental stress						
38- and 50-year-olds	0.96 (0.58-1.58)	0.87	1.00 (0.68-1.49)	0.99	1.00 (0.64-1.56)	0.99

No significant interactions between year and SEP were detected

### Smoking, BMI and leisure time physical activity

The trend showed a decreased proportion of women who smoked (*p* < 0.01) from 38% in 1980 to 9% in 2016 (concerning 38-year-old women) (Table 1). The proportion of women with overweight and obesity (BMI ≥25) increased significantly from 17% in 1980 to 32% in 2016 (Table 1). Also the proportion of women with high leisure time physical activity increased significantly from 66% in 1980 to 93% in 2016 (Table 1).

## Discussion

Our results showed that the proportion of 50-year-old women who perceived their well-being as good increased significantly from 1980 to 2016. In contrast, no significant differences were seen regarding perception of well-being in 38-year-old women. The percentage of women perceiving high mental stress was significantly higher in 2016 than in 1980 in both 38-and 50-year-old women. Interestingly, later-born 50-year old cohorts experienced increasing well-being over time despite simultaneous increases in stress.

Belonging to a low socio-occupational group was associated with perceived poor well-being in 1980 but not in 2016. In other words, there was a suggestive trend over time, such that low socio-occupational group as a risk factor for poor well-being disappeared from 1980 to 2016. Low socio-occupational group was not a risk factor for high perceived mental stress; no associations were found in 1980, 2004 and in 2016.

It should be noted that the aim was to examine self-reported well-being, rather than mortality and morbidity endpoints. Other studies have clearly demonstrated that subjective self-rated health is a good predictor of use of health care/mortality [26] and metabolic health [27].

The strengths of this study are its long duration and the stability of the examination protocols, which have been maintained over a long period of time (36 years). In particular, the similarity of the questionnaires used by the examining physicians allowed us to compare responses over time to the greatest extent possible. Moreover, the participants were sampled on a population basis, based on specified dates of each birth, and satisfactory participation rates at all survey years improves the generalizability of our observations regarding well-being and mental stress in different SEP groups.

However, one limitation of the study is that the participation rate declined from 1980 to 2016 (Fig. 1), such that 84% of invited women participated in 1980, compared to 59% in 2004 and 68% in 2016. Thus, the time- trends may be over- or under-estimated due to variations in participation. A comparison of participants and non-participants was conducted in 2004, because of the declining participation rate [14]. There were no differences in marital status, hospital admission rate or places of living between participants and non-participants. However, significant differences were observed concerning income and immigration status, with lower mean income and higher proportion of immigrants in the non-participant group. Specifically regarding secular trends in mental stress, it should be noted that the concept of mental stress may have changed or become more normalized between 1980 and 2016, and could explain in part some of the increases. Even in 1980, the observed rates were much higher than in the late 1960’s, when the concept of mental stress was less popularized.

Our results on secular trends in well-being are to a large extent consistent with another longitudinal study from SALLS (Swedish Annual Level of Living Survey) [16]. Improved self-rated health was seen in men and women, aged > 48 years between 1980 and 2004, but self-rated health became poorer or was unchanged in those aged 16–47. Moreover, in the MONICA study in northern Sweden [28], women’s self-rated health declined from 1990 to 2014 whereas men’s self-rated health increased. A French study showed a general decline in health-related quality of life between 1995 and 2016 in the female population [29]. Sleep problems, identified as an important stressor, also have substantial impact on health and well-being [30]. Sleep problems were included as stress factor in our mental stress question, and thus included as mental stress augmenter. In contrast, sleep problems were not named explicitly in the well-being question.

The increase of the proportion of women who experience high mental stress is also seen in other studies. Despite the fact that Sweden is an equal country, there are large differences in the number of unpaid working hours (women 26.5 h/week, men 21 h/week) [31]. Life expectancy is increasing in Sweden, but clear differences remain. Health gaps based on education are growing. Women with low level of education have only marginally increased their life expectancy, while survival rates have increased among women with high level of education [32]. The results of the present study are not entirely consistent with this.

Several possible explanations for the difference in well-being between Swedish 38- and 50-year-old women are possible. Today’s 38-year-old women are more likely to have young children, compared to previous generations. This is related to women having a higher average age at the first marriage [33]. In 1980, the average age at first marriage in Sweden was 26. In 2004, the average age at first marriage was 32 and in 2016, the average age was 34. Another possible influence is a higher median age for the first divorce, from 36 years in 1980 to 42 years in 2014 [33]. Finally, changes in BMI represent another possible explanation for the difference in well-being between 38- and 50-year-old women observed in the present study. While both 38-and 50-year-old cohorts reported changes in smoking and physical activity, only the 38-year-old cohorts had an increasing significant trend showing an increase in the prevalence of BMI ≥25, whereas in the 50-year-old cohorts prevalences were unchanged.

## Conclusions

Middle aged women of today seem to perceive both high well-being and high mental stress irrespective of belonging to low or high SEP group. This is in contrast to 1980, when poor well-being was associated with low SEP. Significant secular trends were seen, such that low SEP as risk factor for poor well-being disappeared from 1980 to 2016. Perception of high mental stress increased between 1980 and 2016, for both 38-and 50-year-old women, irrespective of SEP. Further studies are needed to explore determinants of women’s well-being and mental stress, including studies within the context of the ongoing global pandemic.

## Data Availability

The datasets used and analysed during the current study are not publicly available due to Swedish law, but are available from the corresponding author on reasonable request. The database is partly described in Swedish National Dataservice https://snd.gu.se/en/catalogue/study/snd0009#description

## Abbreviations

SEP: Socioeconomic Position
PPSWG: Prospective Population Study of Women in Gothenburg
GQL: Gothenburg Quality of Life
WHO: World Health Organization
BMI: Body Mass Index
OR: Odds Ratios
CI: Confidence Intervals
SAS: Statistical Analysis System
SALLS: Swedish Annual Level of Living Survey
